# Protective Effects of [6]-Gingerol Against Chemical Carcinogens: Mechanistic Insights

**DOI:** 10.3390/ijms21030695

**Published:** 2020-01-21

**Authors:** Veronika Furlan, Urban Bren

**Affiliations:** 1 Faculty of Chemistry and Chemical Technology, University of Maribor, Smetanova 17, SI-2000 Maribor, Slovenia; veronika.furlan@um.si; 2 National Institute of Chemistry, Hajdrihova 19, SI-1001 Ljubljana, Slovenia

**Keywords:** [6]-gingerol, glutathione, chemical carcinogens of the epoxy type, carcinogenesis, activation free energies, quantum-mechanical calculations

## Abstract

[6]-Gingerol from ginger has received considerable attention as a potential cancer therapeutic agent because of its chemopreventive and chemotherapeutic effects, as well as its safety. In the current study, we examined [6]-gingerol as a natural scavenger of nine ultimate chemical carcinogens to which we are frequently exposed: glycidamide, styrene oxide, aflatoxin B1 exo-8,9-epoxide, *β*-propiolactone, ethylene oxide, propylene oxide, 2-cyanoethylene oxide, chloroethylene oxide, and vinyl carbamate epoxide. To evaluate [6]-gingerol efficacy, we expanded our research with the examination of glutathione—the strongest natural scavenger in human cells. The corresponding activation free energies were calculated using Hartree-Fock method with three flexible basis sets and two implicit solvation models. According to our results, [6]-gingerol proves to be an extremely effective scavenger of chemical carcinogens of the epoxy type. On the other hand, with the exception of aflatoxin B1 exo-8,9-epoxide, glutathione represents a relatively poor scavenger, whose efficacy could be augmented by [6]-gingerol. Moreover, our quantum mechanical study of the alkylation reactions of chemical carcinogens with [6]-gingerol and glutathione provide valuable insights in the reaction mechanisms and the geometries of the corresponding transition states. Therefore, we strongly believe that our research forms a solid basis for further computational, experimental and clinical studies of anticarcinogenic properties of [6]-gingerol as well as for the development of novel chemoprophylactic dietary supplements. Finally, the obtained results also point to the applicability of quantum chemical methods to studies of alkylation reactions related to chemical carcinogenesis.

## 1. Introduction

Cancer is a major cause of death in developed countries, second after cardiac diseases. Carcinogenesis represents a complex pathological process where normal cells become neoplastic. In most of the cases, carcinogenesis is associated with chemical modification of DNA. If the harmful chemicals come from the environment, they are referred to as exogenous chemical carcinogens. Nowadays, exogenous chemical carcinogens are indeed implicated in the etiology of an increasing number of cancers. The focus of the current study was to examine [6]-gingerol from ginger and glutathione as natural scavengers of nine ultimate chemical carcinogens of the epoxy type to which we are frequently exposed: glycidamide, styrene oxide, aflatoxin B1 exo-8,9-epoxide, *β*-propiolactone, ethylene oxide, propylene oxide, 2-cyanoethylene oxide, chloroethylene oxide, and vinyl carbamate epoxide.

### 1.1. Chemical Carcinogens

Acrylamide is of great biological interest due to its role in the etiology of cancer. Its presence in a variety of fried and oven-cooked foods is not negligible and can amount to 100 µg per day [1,2]. Acrylamide is metabolized in vivo through epoxidation by cytochrome P450 2E1 [3] to glycidamide. Glycidamide represents the ultimate carcinogen of acrylamide, because it alkylates DNA mainly at the N7 position of guanine leading to the formation of the N7-(2-carbamoyl-2-hydroxyethyl) guanine DNA adduct [1].

Styrene is used in the chemical industry for the production of polystyrene as well as unsaturated polyesters. After absorption through skin or respiration, styrene is metabolized via P450 2E1 into styrene-7,8-oxide (STO) [4]. STO is a direct alkylating agent, which can react with nucleophilic sites in DNA in particular with guanine at position N7 [4,5].

Aflatoxin B1 (AFB1) is among the most potent mutagens implicated in human carcinogenesis. This mycotoxin is produced by the common molds *Aspergillus flavus*, *Aspergillus parasiticus*, and *Aspergillus nomius* which infest agricultural commodities stored in hot moist conditions [6]. AFB1 is primarily metabolized in humans by cytochrome P450 3A4 to yield the ultimate carcinogen AFB1 exo-8,9-epoxide [7]. This very reactive electrophile alkylates DNA with high regiospecificity at the N7 position of guanine, yielding the trans-8,9-dihydro-8-(N7-guanyl)-9-hydroxyaflatoxin adduct [6,7].

Beta-propiolactone (BPL) represents a possible human carcinogen, which is used in vaccines for the inactivation of viruses [8]. Carcinogenicity of beta-propiolactone is strongly connected with its reactivity as a monoalkylating agent. The reactions of nucleophilic centers on DNA with BPL yield 7-(2-carboxyethyl) guanine (7-CEG) as the main product [9].

Polyaromatic hydrocarbons (PAHs) are another family of procarcinogens which are not carcinogenic per se, but are rather metabolized by cytochromes P450 to the highly reactive epoxidized forms called ultimate carcinogens that react with DNA, typically with guanine at position N7 [5]. Ethylene oxide (ETO) represents the smallest compound that can be used to model PAH ultimate carcinogens [10]. It is used for medical equipment sterilization and hospital disinfection. The N7 of guanine is also the major site of ETO alkylation [10].

Propylene represents a procarcinogen which is, after intake, metabolized by the action of cytochrome P450 enzymes to propylene oxide [11]. Propylene and propylene oxide (PO) are used for food sterilization as well as in the production of polyurethane foams, solvents, antifreeze, and resins. PO is also applied as a disinfectant sterilizing agent and a fumigant. Exposition of rats to PO gives rise to an increased incidence of breast carcinoma. PO is a direct alkylating agent that can react with nucleophilic sites in DNA, in particular with guanine at position N7 [11].

Acrylonitrile is industrial monomer used in the synthesis of acrylic fibers, nitrile rubbers, and resins. In the presence of oxygen and NADPH, its double bond can be epoxidized by cytochrome P450 2E1 to yield the 2-cyanoethylene oxide [3]. 2-cyanoethylene oxide represents the ultimate carcinogen of acrylonitrile because it alkylates DNA mainly at the N7 position of guanine leading to the formation of the N^7^-(2-oxoethyl) guanine adduct [12].

Vinyl chloride (VC) represents an exogenous chemical carcinogen, which has been manufactured in large quantities to poly (vinyl chloride) (PVC). VC is also found in small quantities in groundwater and in tobacco smoke [13]. According to *in vitro* [14] and *in vivo* [15] studies, VC is epoxidized by cytochrome P450 2E1 in the presence of oxygen and NADPH to chloroethylene oxide (CEO). The major alkylation site of CEO is the N7 position of guanine, which yields 7-(2-oxyethyl) guanine [13].

Urethane (ethyl carbamate) can be found in food products including yogurt, bread, soy sauce, and cheese, as well as in tobacco and alcoholic beverages. This omnipresence is a consequence of urethane being formed as a byproduct of fermentation [16]. Urethane is after intake in the presence of oxygen and NADPH metabolized by cytochrome P450 2E1 into its ultimate carcinogen vinyl carbamate epoxide (VCE) [17]. This electrophilic species then reacts with DNA, usually by alkylating guanine at the N7 position, leading to the main adduct 7-(2-oxoethyl) deoxyguanosine [16].

All studied ultimate carcinogens are, therefore, electrophilic species which react with DNA, usually by alkylating its most nucleophilic site—guanine at the N7 position, leading to the formation of the ultimate chemical carcinogen-guanine adduct. It is well established that the rate-limiting step for the reaction of the ultimate carcinogens of the epoxy type with the nucleophilic sites of DNA and other nucleophilic molecules is the epoxy ring opening [1]. For this rate-limiting step of all studied alkylation reactions, the *S_N_2* substitution mechanism is proposed. Subsequent protonation is believed to be a fast process due to a proton-rich microenvironment surrounding the negatively charged DNA. Alkylation is then followed by depurination leading to gene mutations and chromosomal aberrations [1].

### 1.2. [6]-Gingerol and Glutathione

Natural dietary agents including fruits, vegetables, and spices consist of a wide variety of biologically active compounds that are responsible for the chemopreventive and chemotherapeutic effects. The molecular mechanisms by which biologically active compounds prevent cancer initiation and progression have been reviewed in a recent article [18].

Ginger (*Zingiber officinale Roscoe*) rhizome is one of the hot spices belonging to the *Zingiberaceae* family native to Southern Asia [19]. The rhizome of the plant has been used in traditional Asian, Indian and Arabic medicine since antiquity to treat rheumatoid arthritis, sprains and muscular aches, sore throats, nausea, constipation and indigestion, fever, infectious diseases as well as inflammation [20]. Currently, one can observe renewed interest in this medicinal spice and investigations involving isolation and identification of its bioactive constituents, as well as experimental validation of their empirical pharmacological actions. Ginger represents an excellent source of several bioactive phenolics, including non-volatile pungent compounds such as gingerols, paradols, shogaols, and zingerones [19]. Phenolic substances present in ginger generally possess strong antiinflammatory and antioxidative properties as well as exert substantial anticarcinogenic and antimutagenic activities [21]. Its major phenolic bioactive constituent [6]-gingerol (1-[40-hydroxy30-methoxyphenyl]-5-hydroxy-3-decanone), an oily liquid and the most prevalent representative of gingerols in the fresh ginger rhizome, has received considerable attention as a potential therapeutic agent because of its efficacy through regulation of multiple biomolecular pathways as well as its safety [19].

**Figure 1:**
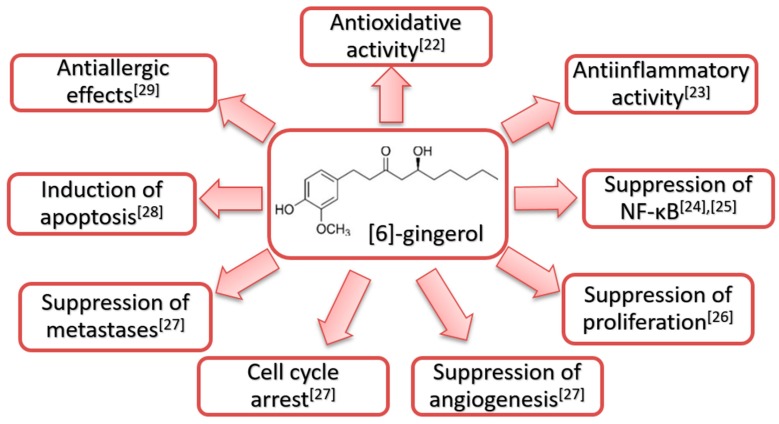
Structural formula and reported biological effects of [6]-gingerol [22,23,24,25,26,27,28,29].

The chemopreventive and chemotherapeutic effects exerted by [6]-gingerol are presented in Figure 1.

As can be observed in Figure 1, numerous mechanisms of chemopreventive and chemotherapeutic effects of [6]-gingerol have been reported in the scientific literature. The results of the study conducted by Radhakrishnan et. al. [28] revealed inhibition of cell proliferation and induction of apoptosis in mouse colon cancer cells, while the normal colon cells remained unaffected. The inhibition of extracellular signal–regulated kinase/c-Jun N-terminal kinase/activator protein 1 (ERK1/2/JNK/AP-1) signaling pathway was reported as a possible mechanism of chemopreventive as well as chemotherapeutic efficacy of [6]-gingerol against colon cancer. The inhibition of cell proliferation through mitogen-activated protein kinase (MAPK) and AP-1 signaling pathways in human skin keratinocyte cell lines exposed to [6]-gingerol was also observed [26]. Moreover, topical application of [6]-gingerol inhibited tetradecanoylphorbol-13-acetate (TPA)-induced COX-2 expression along with suppressed p38 MAPK and nuclear factor kappa B (NF-κB) DNA binding activity in mouse skin [24].

Angiogenesis, the formation of new blood vessels from pre-existing endothelium, represents a fundamental process in tumor development. Kim et. al. [27] reported that [6]-gingerol possesses potential anti-angiogenic activity *in vitro* and *in vivo*. *In vitro*, [6]-gingerol inhibited angiogenesis of human endothelial cells and caused cell cycle arrest in the G1 phase through the down-regulation of cyclin D1. *In vivo*, [6]-gingerol suppressed tumor growth and formation of metastases presumably by inhibition of angiogenesis in tumor-bearing mice. Moreover, [6]-gingerol reduced matrix metalloproteinase 9 (MMP-9) expression in pancreatic cancer cells through ERK/NF-κB/snail signal transduction pathway [30]. These results point towards a potent role of [6]-gingerol in preventing malignant cancer cell growth.

According to the scientific literature, [6]-gingerol proves to be a potent suppressor of promotion as well as the progression of carcinogenesis and therefore possesses a potential to become a multitarget anticancer drug. On the other hand, the potential of [6]-gingerol as a blocking agent, which can prevent the initiation of carcinogenesis triggered by the studied chemical carcinogens, has not been studied in detail yet.

Glutathione (GSH) represents one of the major water soluble biomolecules involved in cellular detoxification processes, protecting the cells against xenobiotic agents generating oxidative stress [31,32,33]. Tripeptide glutathione can scavenge free radicals, reduce peroxides or be conjugated with electrophilic xenobiotics. Conjugation of glutathione to xenobiotics reduces their toxicity and reactivity as well as makes them more polar and prone to excretion. Glutathione, therefore, provides cells with multiple defense mechanisms not only against the reactive oxygen species but also against toxic chemicals [31]. Accordingly, GSH can be regarded as a major factor regulating cell proliferation, differentiation, and apoptosis [32]. Any ultimate carcinogen can, therefore, under physiological conditions react with DNA and one or more scavengers, e.g., glutathione.

**Scheme A1:**
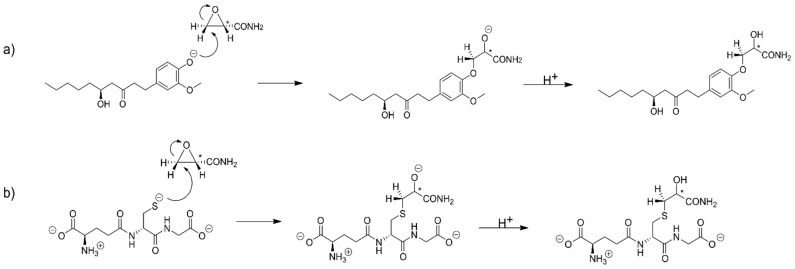
The proposed reaction mechanism of glycidamide with (**a**) [6]-gingerol and (**b**) glutathione.

**Scheme A2:**
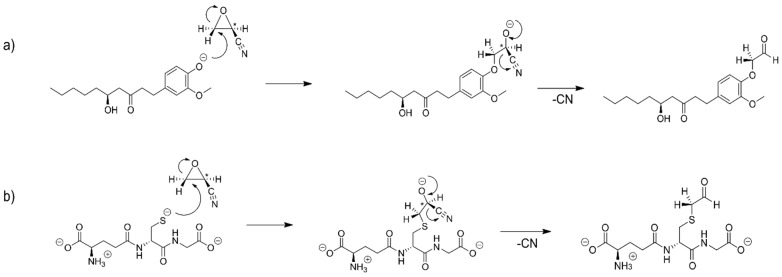
The proposed reaction mechanism of 2-cyanoethylene oxide with (**a**) [6]-gingerol and (**b**) glutathione.

**Scheme A3:**
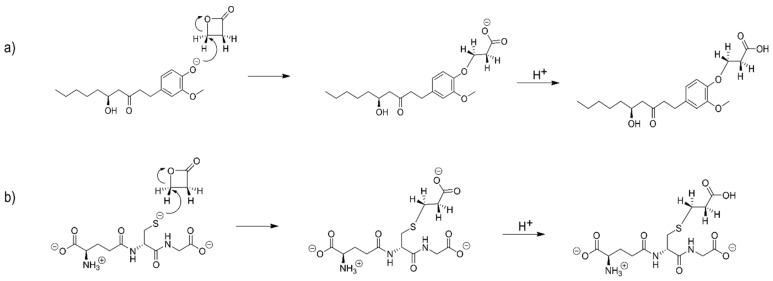
The proposed reaction mechanism of beta-propiolactone with (**a**) [6]-gingerol and (**b**) glutathione.

**Scheme A4:**
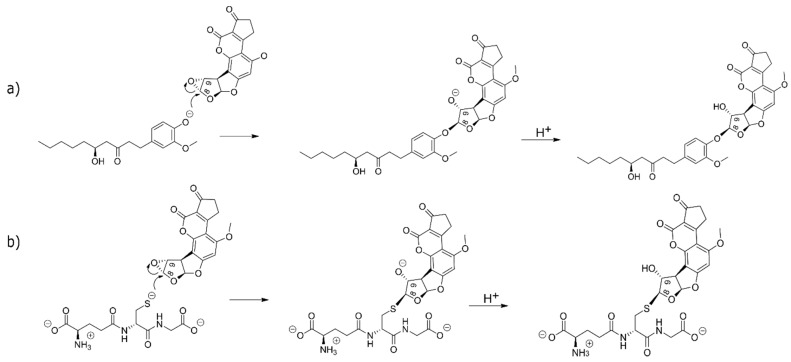
The proposed reaction mechanism of AFB1 exo-8,9-epoxide with (**a**) [6]-gingerol and (**b**) glutathione.

**Scheme A5:**
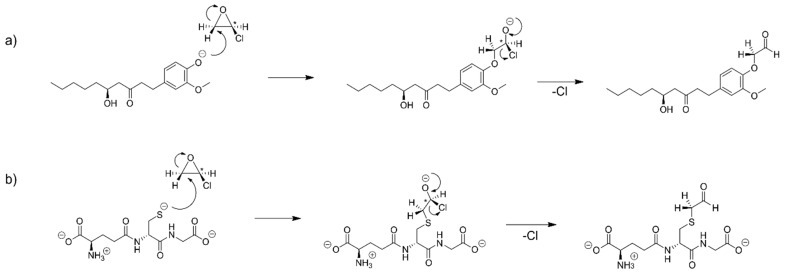
The proposed reaction mechanism of chloroethylene oxide with (**a**) [6]-gingerol and (**b**) glutathione.

**Scheme A6:**
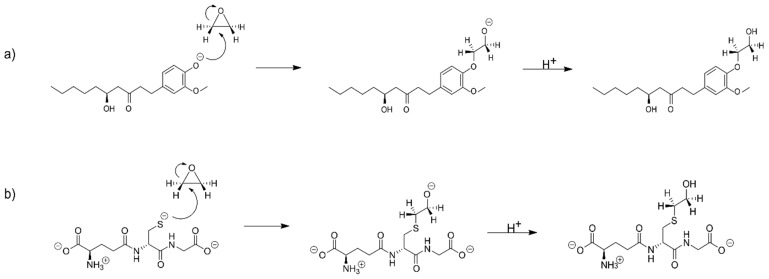
The proposed reaction mechanism of ethylene oxide with (**a**) [6]-gingerol and (**b**) glutathione.

**Scheme A7:**
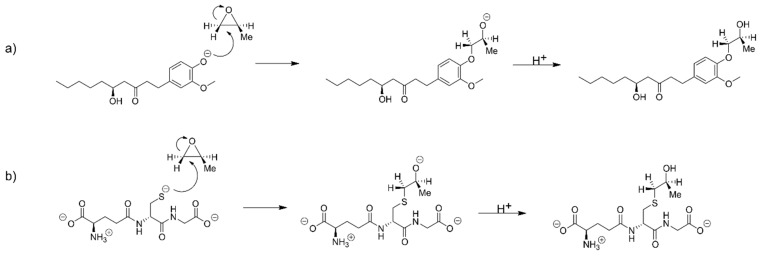
The proposed reaction mechanism of propylene oxide with (**a**) [6]-gingerol and (**b**) glutathione.

**Scheme A8:**
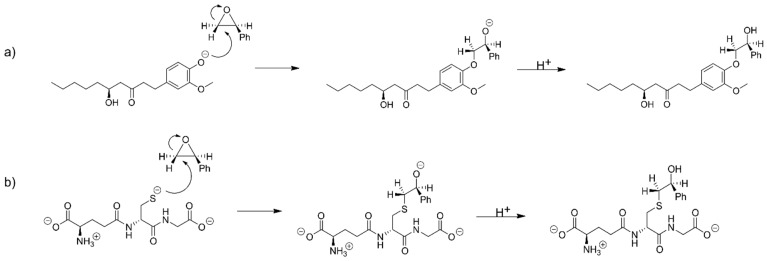
The proposed reaction mechanism of styrene oxide with (**a**) [6]-gingerol and (**b**) glutathione.

**Scheme A9:**
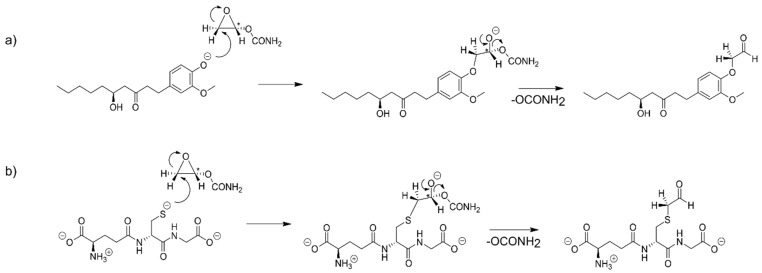
The proposed reaction mechanism of vinyl carbamate epoxide with (**a**) [6]-gingerol and (**b**) glutathione.

Both [6]-gingerol and glutathione were examined in their nucleophilic (anionic) forms at physiological pH of 7.4. In the absence of experimental data, we calculated the pKa values for [6]-gingerol and glutathione with the MarvinSketch software [34]. At physiological conditions, the predicted pKa value for the phenol group of [6]-gingerol was slightly lower (by 0.1) than for the thiol group of glutathione resulting in a 1% higher abundance of simulated ionic species in the case of [6]-gingerol. The proposed *S_N_2* substitution mechanisms for the formation of the chemical carcinogen-[6]-gingerol adducts are depicted in Scheme A1, Scheme A2, Scheme A3, Scheme A4, Scheme A5, Scheme A6, Scheme A7, Scheme A8 and Scheme A9 available in the Appendix A. Similarly, the *S_N_2* substitution mechanisms are proposed for the formation of ultimate carcinogen-glutathione adducts, which are depicted in Scheme A1, Scheme A2, Scheme A3, Scheme A4, Scheme A5, Scheme A6, Scheme A7, Scheme A8 and Scheme A9 available in the Appendix A as well. The proposed molecular mechanisms in Scheme A1, Scheme A2, Scheme A3, Scheme A4, Scheme A5, Scheme A6, Scheme A7, Scheme A8 and Scheme A9 were drawn using the ChemDraw program.

**Figure 2:**
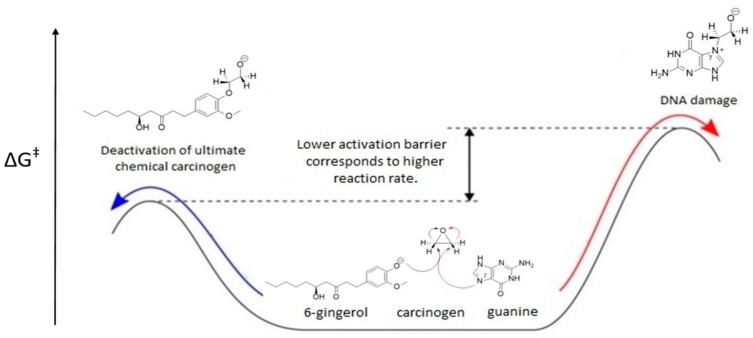
Activation free energy ∆G^⧧^ for the reaction between the chemical carcinogen and natural scavenger (blue arrow) in comparison with activation free energy for competing reaction between the chemical carcinogen and guanine (red arrow).

The focus of the current contribution was to examine [6]-gingerol from ginger as a polyphenolic scavenger of nine ultimate chemical carcinogens of the epoxy type: glycidamide, styrene oxide, aflatoxin B1 exo-8,9-epoxide, *β*-propiolactone, ethylene oxide, propylene oxide, 2-cyanoethylene oxide, chloroethylene oxide, and vinyl carbamate epoxide. To evaluate [6]-gingerol efficacy, we expanded our research by introducing glutathione—the strongest natural scavenger of chemical carcinogens in human cells. This is the first study that addresses the kinetics of ultimate carcinogen-[6]-gingerol and ultimate carcinogen-glutathione adduct formation by focusing on ΔG^⧧^, the activation free energy of the rate-limiting step of these alkylation reactions. This quantity is directly related to the overall reaction rate and thus to the carcinogenicity of nine studied chemical carcinogens [13]. Lower activation barrier for the reaction between natural scavenger and chemical carcinogen implies that this reaction will be faster than the competing reaction between the chemical carcinogen and the most nucleophilic DNA base guanine with higher activation barrier (Figure 2). Consequently, such natural scavengers can efficiently protect DNA from alkylation with chemical carcinogens and prevent cancer initiation.

## 2. Results and Discussion

Our in silico calculations focus on the first step of the reaction between the chemical carcinogen and natural scavenger, namely [6]-gingerol or glutathione, as this proposed *S_N_2* substitution represents the rate-limiting step for all reactions. The activation free energy is defined as the free energy difference between the transition state and the reactants. To obtain the activation free energy of this *S_N_2* substitution, we considered free energies of reactants and transition state (the saddle point characterized by a single imaginary frequency) on the potential energy surface of the corresponding reaction coordinate [1,7].

**Figure 3:**
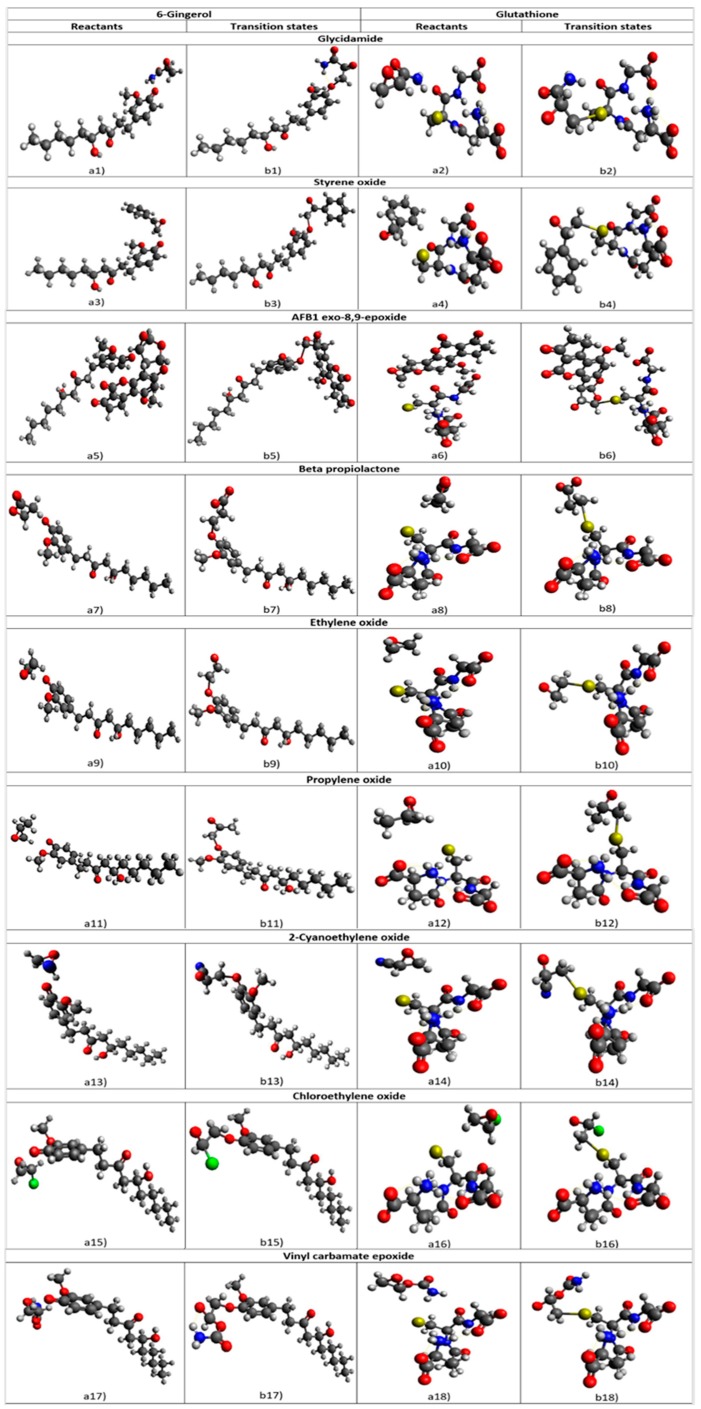
The structures of the reactant states (**a1**–**a18**) and the transition states (**b1**–**b18**) for the nucleophilic attack of the phenolic oxygen atom of [6]-gingerol and the sulfur atom of glutathione through the alkylation reaction with the nine studied ultimate chemical carcinogens as predicted by the Hartree-Fock (HF) method using the 6-311++G(d,p) basis set. Carbon atoms are depicted in gray, oxygen atoms in red, nitrogen atoms in blue, sulfur atoms in yellow and hydrogen atoms in white color.

Structures of reactants and transition states corresponding to the eighteen studied alkylation reactions between nine ultimate chemical carcinogens and 6-gingerol as well as glutathione are presented in Figure 3.

Graphical representations of the obtained results reveal the formation of the chemical bond between the phenolic oxygen atom of [6]-gingerol or sulfur atom of glutathione and the nonchiral carbon in the epoxy ring of all studied ultimate chemical carcinogens. The simultaneous cleavage of the chemical bond connecting this nonchiral carbon of the ultimate chemical carcinogen to its epoxy oxygen confirmed the allocation of the correct transition state structure. The exclusively obtained real frequencies for all reactant state structures and a single imaginary frequency with its normal mode corresponding to such reactive process obtained for all transition state structures present strong evidence in favor of the validity of the proposed *S_N_2* reaction mechanism.

The calculated activation barriers at the HF/6-311++G(d,p) level of theory for the alkylation reactions between the nine studied chemical carcinogens and [6]-gingerol as well as glutathione *in vacuo* and solvated using the Self-consistent reaction field (SCRF) method, imaginary frequencies of transition states, lowest vibrational frequencies of reactant states and corresponding distances between the reactive centers are collected in Table 1. Table S1 with the corresponding computational results obtained at the Hartree-Fock level of theory with three different flexible basis sets in conjunction with SCRF and Langevin dipoles (LD) implicit solvation models can be found in the Supplementary Material.

### 2.1. The Alkylation Reaction of Glycidamide with [6]-Gingerol and Glutathione

The structures of the reactants (a1,a2) and the transition states (b1,b2) for the alkylation of glycidamide with [6]-gingerol and glutathione, respectively, are presented in Figure 3.

For the reaction with [6]-gingerol the computed gas-phase activation barrier at the Hartree-Fock level of theory in conjunction with three flexible basis sets lies between 30 kcal/mol and 32 kcal/mol. The computed gas-phase activation barrier at the same level of theory for the reaction with glutathione is higher laying between 35 kcal/mol and 39 kcal/mol (Table S1). The predicted activation free energy with the SCRF method at the HF/6-311++G(d,p) level of theory is 25.23 kcal/mol for reaction with [6]-gingerol. Using identical methodology predicted activation free energy for the reaction with glutathione is significantly (~7 kcal/mol) higher (Table 1). The corresponding distance between the reactive centers of the reactants is approximately 0.94 Å shorter for the reaction with [6]-gingerol than for the reaction with glutathione. The reacting molecules in the transition states are approximately 0.5 Å closer for the reaction with [6]-gingerol as well reflecting the larger van der Waals radius of the nucleophilic sulfur in glutathione. The reduction in the activation free energy is approximately −5.6 kcal/mol for the reaction with [6]-gingerol and is significantly stronger (by −2 kcal/mol) than for the reaction with glutathione (Table 1). Therefore, transition states with glycidamide are relatively better hydrated in the reactions with [6]-gingerol, while reactants are relatively better hydrated in the reactions with glutathione.

### 2.2. The Alkylation Reaction of Styrene Oxide with [6]-Gingerol and Glutathione

The structures of the reactants (a3,a4) and the transition states (b3,b4) for the alkylation of styrene oxide with [6]-gingerol and glutathione, respectively, are presented in Figure 3.

For the reaction with [6]-gingerol, the computed gas-phase activation barrier obtained using the HF method with three flexible basis sets lies between 26 kcal/mol and 27 kcal/mol. The analogously computed gas-phase activation barrier for the reaction with glutathione is higher and lies between 30 kcal/mol and 34 kcal/mol (Table S1). The predicted activation free energy using SCRF method in conjunction with the HF/6-311++G(d,p) level of theory is 21.65 kcal/mol for the reaction with [6]-gingerol, which is significantly lower (~10 kcal/mol) than for the reaction with glutathione (Table 1). The corresponding distance between the reactive centers of the reactants is approximately 1 Å shorter for the reaction with [6]-gingerol than for the reaction with glutathione. The reacting molecules in the transition state are ~0.5 Å closer for the reaction with [6]-gingerol as well reflecting the larger van der Waals radius of the nucleophilic sulfur in glutathione. The reduction in the activation free energy is approximately −4 kcal/mol for the reaction with [6]-gingerol and is significantly stronger (by ~−2,4 kcal/mol) than for the reaction with glutathione (Table 1). Transition states with styrene oxide are therefore relatively better hydrated in the reactions with [6]-gingerol, while reactants are relatively better hydrated in the reactions with glutathione.

### 2.3. The Alkylation Reaction of AFB1 Exo-8,9-Epoxide with [6]-Gingerol and Glutathione

The structures of the reactants (a5, a6) and the transition states (b5, b6) for the alkylation of AFB1 exo-8,9-epoxide with [6]-gingerol and glutathione, respectively, are presented in Figure 3.

For the reaction with [6]-gingerol, the computed gas-phase activation barrier obtained using the HF method with three flexible basis set lies between 18 kcal/mol and 21 kcal/mol. The analogously predicted gas-phase activation barrier for the reaction with glutathione is similar and lies between 17 kcal/mol and 21 kcal/mol (Table S1). The predicted activation free energy using HF/6-311++G(d,p) method in conjunction with SCRF solvation model is 15.38 kcal/mol for the reaction with [6]-gingerol, which is only 0.3 kcal/mol lower than for the reaction with glutathione (Table 1). The corresponding distance between the reactive centers of the reactants is approximately 0.4 Å shorter for the reaction with [6]-gingerol than for the reaction with glutathione. The reacting molecules in the transition state are ~0.4 Å closer for the reactions with [6]-gingerol as well reflecting the larger van der Waals radius of the nucleophilic sulfur in glutathione. The reduction in the activation free energy is approximately −5.5 kcal/mol for the reaction with [6]-gingerol and is significantly stronger (by ~ −3.6 kcal/mol) than for the reaction with glutathione (Table 1). Consequently, transition states with AFB1 exo-8,9-epoxide are relatively better hydrated in the reactions with [6]-gingerol, while reactants are relatively better hydrated in the reactions with glutathione.

### 2.4. The Alkylation Reaction of β-Propiolactone with [6]-Gingerol and Glutathione

The structures of the reactants (a7,a8) and the transition states (b7,b8) for the alkylation of *β*-propiolactone with [6]-gingerol and glutathione, respectively, are presented in Figure 3.

For the reaction with [6]-gingerol, the computed gas-phase activation barrier at the Hartree-Fock level of theory with three flexible basis sets lies between 15 kcal/mol and 17 kcal/mol. The analogously computed gas-phase reaction barrier for the reaction with glutathione is higher and lies between 22 kcal/mol and 25 kcal/mol (Table S1). The predicted activation free energy using SCRF method at the HF/6-311++G(d,p) level of theory corresponds to 14,93 kcal/mol for the reaction with [6]-gingerol, which is significantly lower (by ~8 kcal/mol) than for the reaction with glutathione (Table 1). The corresponding distance between the reactive centers of the reactants is approximately 1.0 Å shorter for the reaction with [6]-gingerol than for the reaction with glutathione. The reacting molecules in the transition states are 0.5 Å closer for the reaction with [6]-gingerol as well reflecting the larger van der Waals radius of the nucleophilic sulfur in glutathione. The reduction in the activation free energy is approximately −1.6 kcal/mol for the reaction with [6]-gingerol and is somewhat stronger (by ~ −1.2 kcal/mol) than for the reaction with glutathione (Table 1). Therefore, transition states with *β*-propiolactone are relatively better hydrated in the reactions with [6]-gingerol, while reactants are relatively better hydrated in the reactions with glutathione.

### 2.5. The Alkylation Reaction of Ethylene Oxide with [6]-Gingerol and Glutathione

The structures of the reactants (a9,a10) and the transition states (b9,b10) for the alkylation of ethylene oxide with [6]-gingerol and glutathione, respectively, are presented in Figure 3.

For the reaction with [6]-gingerol the computed gas-phase activation barrier obtained using the HF method with three flexible basis sets lies between 23 kcal/mol and 25 kcal/mol. The analogously computed gas-phase activation barrier for the reaction with glutathione is higher and lies between 30 kcal/mol in 36 kcal/mol (Table S1). The predicted activation free energy obtained using HF/6-311++G(d,p) method in conjunction with SCRF solvation model is 24.03 kcal/mol for the reaction with [6]-gingerol, which is ~6 kcal/mol lower than for the reaction with glutathione (Table 1). The corresponding distance between the reactive centers of the reactants is approximately 0.9 Å shorter for the reaction with [6]-gingerol than for the reaction with glutathione. The reacting molecules in the transition state are ~0.5 Å closer for the reaction with [6]-gingerol as well reflecting the larger van der Waals radius of the nucleophilic sulfur in glutathione. The reduction in the activation free energy is approximately −0.5 kcal/mol for the reaction with [6]-gingerol and is somewhat weaker (by ~0.2 kcal/mol) than for the reaction with glutathione (Table 1). Consequently, transition states with ethylene oxide are relatively better hydrated in the reactions with glutathione, while reactants are relatively better hydrated in the reactions with [6]-gingerol.

### 2.6. The Alkylation Reaction of Propylene Oxide with [6]-Gingerol and Glutathione

The structures of the reactants (a11,a12) and the transition states (b11,b12) for the alkylation of propylene oxide with [6]-gingerol and glutathione, respectively, are presented in Figure 3.

For the reaction with [6]-gingerol the computed gas-phase activation barrier at the Hartree-Fock level of theory with three flexible basis sets lies between 24 kcal/mol and 26 kcal/mol. The analogously computed gas-phase activation barrier for the reaction with glutathione is higher and lies between 31 kcal/mol and 36 kcal/mol (Table S1). The predicted activation free energy obtained using HF/6-311++G(d,p) method in conjunction with SCRF solvation model is 24.37 kcal/mol for the reaction with [6]-gingerol, which is ~6 kcal/mol lower than for the reaction with glutathione (Table 1). The corresponding distance between the reactive centers of the reactants is approximately 0.8 Å shorter for the reaction with [6]-gingerol than for the reaction with glutathione. The reacting molecules in the transition state are ~0.5 Å closer for the reaction with [6]-gingerol as well reflecting the larger van der Waals radius of the nucleophilic sulfur in glutathione. The reduction in the activation free energy is only −0.04 kcal/mol for the reaction with [6]-gingerol and is somewhat weaker (by ~1 kcal/mol) than for the reaction with glutathione (Table 1). Consequently, transition states with propylene oxide are relatively better hydrated in the reaction with glutathione, while reactants are relatively better hydrated in the reactions with [6]-gingerol.

### 2.7. The Alkylation Reaction of 2-Cyanoethylene Oxide with [6]-Gingerol and Glutathione

The structures of the reactants (a13, a14) and the transition states (b13, b14) for the alkylation of 2-cyanoethylene oxide with [6]-gingerol and glutathione, respectively, are presented in Figure 3.

For the reaction with [6]-gingerol, the computed gas-phase activation barrier obtained with the HF method and three flexible basis sets lies between 19 kcal/mol and 21 kcal/mol. The analogously computed gas-phase activation barrier for the reaction with glutathione is higher and lies between 26 kcal/mol and 27 kcal/mol (Table S1). The predicted activation free energy obtained using HF/6-311++G(d,p) method in conjunction with SCRF solvation model is 20.27 kcal/mol for the reaction with [6]-gingerol, which is by ~4 kcal/mol lower than for the reaction with glutathione (Table 1). The corresponding distance between the reactive centers of the reactants is 0.70 Å shorter for the reaction with [6]-gingerol than for the reaction with glutathione. The reacting molecules in the transition states are 0.5 Å closer for the reaction with [6]-gingerol as well reflecting the larger van der Waals radius of the nucleophilic sulfur in glutathione. The reduction in the activation free energy is approximately −0.5 kcal/mol for the reaction with [6]-gingerol and is significantly weaker (by 2 kcal/mol) than for the reaction with glutathione (Table 1). Consequently, transition states with 2-cyanoethylene oxide are relatively better hydrated in the reactions with glutathione, while reactants are relatively better hydrated in the reactions with [6]-gingerol.

### 2.8. The Alkylation Reaction of Chloroethylene Oxide with [6]-Gingerol and Glutathione

The structures of the reactants (a15,a16) and the transition states (b15,b16) for the alkylation of chloroethylene oxide with [6]-gingerol and glutathione, respectively, are presented in Figure 3.

For the reaction with [6]-gingerol the computed gas-phase activation barrier at the Hartree-Fock level of theory using three flexible basis sets lies between 20 kcal/mol and 22 kcal/mol. The analogously computed gas-phase activation barrier for the reaction with glutathione is somewhat higher and lies between 21 kcal/mol and 24 kcal/mol (Table S1). The predicted activation free energy using SCRF method in conjunction with HF/6-311++G(d,p) level of theory is 18.77 kcal/mol for the reaction with [6]-gingerol, which is significantly lower (by ~3 kcal/mol) than for the reaction with glutathione. (Table 1). The corresponding distance between the reactive centers of the reactants is approximately 0.7 Å shorter for the reaction with [6]-gingerol than for the reaction with glutathione. The reacting molecules in the transition state are ~0.5 Å closer for the reactions with [6]-gingerol as well reflecting the larger van der Waals radius of the nucleophilic sulfur in glutathione. The reduction in the activation free energy is approximately −2.4 kcal/mol for the reaction with [6]-gingerol and is significantly stronger (by ~ −2.3 kcal/mol) than for the reaction with glutathione (Table 1). Transition states with chloroethylene oxide are therefore relatively better hydrated in the reactions with [6]-gingerol, while reactants are relatively better hydrated in the reactions with glutathione.

### 2.9. The Alkylation Reaction of Vinyl Carbamate Epoxide with [6]-Gingerol and Glutathione

The structures of the reactants (a17,a18) and the transition states (b17,b18) for the alkylation of vinyl carbamate epoxide with [6]-gingerol and glutathione, respectively, are presented in Figure 3.

For the reaction with [6]-gingerol the computed gas-phase activation barrier at the Hartree-Fock level of theory with three flexible basis sets lies between 18 kcal/mol and 20 kcal/mol. The analogously computed gas-phase activation barrier for the reaction with glutathione is higher and lies between 24 kcal/mol and 26 kcal/mol (Table S1). The predicted activation free energy obtained using HF/6-311++G(d,p) method in conjunction with SCRF solvation model is 18.97 kcal/mol for the reaction with [6]-gingerol, which is ~5.6 kcal/mol lower than for the reaction with glutathione (Table 1). The corresponding distance between the reactive centers of the reactants is ~1.8 Å shorter for the reaction with [6]-gingerol than for the reaction with glutathione. The reacting molecules in the transition state are ~0.4 Å closer for the reaction with [6]-gingerol as well reflecting the larger van der Waals radius of the nucleophilic sulfur in glutathione. The reduction in the activation free energy is approximately −0.2 kcal/mol for the reaction with [6]-gingerol and is somewhat weaker (by ~0.5 kcal/mol) than for the reaction with glutathione (Table 1). Therefore, transition states with vinyl carbamate epoxide are relatively better hydrated in the reactions with glutathione, while reactants are relatively better hydrated in the reactions with [6]-gingerol.

### 2.10. General Remarks

From the acquired gas-phase, SCRF and LD activation barriers collected in Table 1 and Table S1, it is evident that the convergence in terms of basis set size was reached for all ultimate chemical carcinogens. Variation among acquired structures at different flexible basis sets was relatively low as can be observed through highly similar distances between the reactive species. Relative hydration free energies ΔΔG_hydr_ are reported to be negative in all cases, meaning that the transition state is better solvated than the reactant state. Consequently, the solvent lowers the activation barrier and thus accelerates the alkylation reaction. This can be understood in the light of a more dispersed negative charge in the case of phenolic oxygen of [6]-gingerol or of nucleophilic sulfur of glutathione when compared to the opened epoxy oxygen of the corresponding transition state structures.

Larger values of d^R^ compared to d^TS^ are consistent with the much weaker intermolecular interactions in the reactant state and consequently with the much shallower potential hypersurface. Furthermore, a single imaginary vibrational frequency was obtained for the transition state structure with all basis sets (Table 1 and Table S1). Normal modes of these imaginary frequencies were visualized by MOLDEN [35] and Avogardo [36] programs because they should correspond to the reaction coordinate of the first step of the reaction mechanism depicted in Scheme A1, Scheme A2, Scheme A3, Scheme A4, Scheme A5, Scheme A6, Scheme A7, Scheme A8 and Scheme A9. For all applied basis sets this normal mode indeed coincided with the formation of a chemical bond between the phenolic oxygen of [6]-gingerol or nucleophilic sulfur of glutathione and the nonchiral carbon of the epoxy group on the chemical carcinogen. The simultaneous cleavage of the chemical bond connecting this nonchiral carbon to the epoxy oxygen confirmed the allocation of the correct transition state structure. Obtained results are, therefore, in agreement with the proposed *S_N_2* reaction mechanism.

### 2.11. The Comparison of Activation Free Energies Obtained with Implicit Solvation Models for Alkylation Reactions of the Studied Chemical Carcinogens with [6]-Gingerol and Glutathione

As the examined alkylation reactions take place in a solution, the values of ΔE^⧧^, which are calculated *in vacuo*, cannot be readily compared to the experimental results of kinetic studies [16]. Therefore, we performed a series of calculations where implicit solvation effects were incorporated. In all cases, simulated reactions were initiated from a close-contact reactant configuration trapped within a cage of implicit solvent. Such reactions are unimolecular in nature, and the calculated activation free energies can be, therefore, directly compared to the experimentally measured activation free energies for the first order reactions of chemical carcinogens with their studied natural scavengers or the most nucleophilic DNA base guanine.

As the addition of diffuse and polarization functions on heavy and light atoms is crucial for obtaining the most accurate activation free energies in terms of the basis set size, we computed the activation free energies for reactions of the studied chemical carcinogens with [6]-gingerol, glutathione and guanine at the Hartree-Fock level of theory in conjunction with flexible 6-311++G(d,p) basis set. Table 2 summarizes the results obtained by the SCRF and LD implicit solvation models at the HF/6-311++G(d,p) level of theory for reactions of [6]-gingerol, glutathione and guanine with the studied chemical carcinogens. For a critical evaluation of computational results, the experimentally obtained activation free energies for reactions of the studied chemical carcinogens with guanine are also presented. To determine which solvation model outperforms the other for a given ultimate chemical carcinogen, a comparison of the computed and the experimentally obtained activation free energies for the corresponding guanine alkylation was performed. Furthermore, the global hybrid functional with 54% HF exchange, M06-2X, was used to demonstrate that modern exchange-correlation energy density functionals produce the same relative order of activation free energies, albeit at lower values, which are collected in Table S2.

The Hartree−Fock level of theory in combination with the flexible 6-311++G(d,p) basis set and the LD implicit solvation model was found to better reproduce the experimental activation free energies for the reactions of styrene oxide, propylene oxide, ethylene oxide, glycidamide, 2-cyanoethylene oxide and AFB1 exo-8,9-epoxide with guanine, which could be also expected for the reactions of these chemical carcinogens with [6]-gingerol as well as glutathione. On the other hand, the Hartree−Fock level of theory in combination with the flexible 6-311++G(d,p) basis set and the SCRF implicit solvation model was found to better reproduce the experimental activation free energies of the reactions between vinyl carbamate epoxide, *β*-propiolactone and chloroethylene oxide and guanine, which could then be assumed for the reactions of these chemical carcinogens with [6]-gingerol and glutathione as well.

From Table 2, it is also evident that the quantum mechanical calculations at the HF/6-311++G(d,p) level of theory in conjunction with the implicit solvation model that gives the best agreement with the experiment predicted significantly lower activation free energies of all studied chemical carcinogens with [6]-gingerol than the corresponding experiments between the most reactive DNA nucleobase guanine and these chemical carcinogens. On the other hand, the calculated activation free energies for the reactions between glutathione and seven chemical carcinogens, namely *β*-propiolactone, 2-cyanoethylene oxide, chloroethylene oxide, glycidamide, propylene oxide, styrene oxide and vinyl carbamate epoxide, are higher than the experimentally obtained activation free energies for the competing reactions with guanine. The only exceptions are ethylene oxide and AFB1 exo-8,9-epoxide. Moreover, the quantum mechanical calculations at the same level of theory also predicted a significantly higher reactivity of eight studied chemical carcinogens towards [6]-gingerol than towards glutathione. The only exception is the alkylation reaction with AFB1 exo-8,9-epoxide, where [6]-gingerol and glutathione performed equally well.

On the basis of the calculations at the HF/6-311++G(d,p) level of theory in conjunction with the LD implicit solvation model, the activation free energy is the highest for the reaction of styrene oxide with [6]-gingerol (26.03 kcal/mol) and glutathione (30.16 kcal/mol), respectively. The reaction of styrene oxide with [6]-gingerol and glutathione is, therefore, the slowest. On the other hand, the activation free energy for the reaction of AFB1 exo-8,9-epoxide, the most genotoxic chemical carcinogen with the lowest calculated (14.3 kcal/mol) and experimentally determined (15.1 kcal/mol) free energy barrier towards guanine, with [6]-gingerol (5.44 kcal/mol) and glutathione (5.21 kcal/mol) is the lowest. The reaction of AFB1 exo-8,9-epoxide with [6]-gingerol and glutathione is, therefore, the fastest.

As both SCRF and LD implicit solvation models in conjunction with the HF/6-311++G(d) level of theory provide a good agreement with experimental data, we cannot make a final decision as to which solvation model performs better. However, in contrast to the SCRF model, the Langevin dipoles do to a certain extent involve thermal averaging and specific interactions between solute and solvent. Therefore, the reduction of the activation barriers in terms of the relative hydration free energies is more pronounced using LD than using the SCRF implicit solvation model.

## 3. Computational Methods

The Hartree−Fock (HF) level of theory, combined with flexible 6-311++G(d,p) basis set and implicit solvation model, was found to give a very good agreement with the experimental activation free energies for the alkylation reactions between all nine studied ultimate chemical carcinogens and the most reactive DNA base guanine [1,7,12,13,16]. Moreover, an observation has been reported in the scientific literature, that both the B3LYP density functional and the MP2 theory levels, which include a certain degree of dynamical electron correlation, exhibit moderate to significant underestimation of the experimental ΔG^⧧^ regardless of the applied solvation model [1,7,12,13,16]. Hartree-Fock level of theory also provides a strong argument in favor of the proposed *S_N_2* reaction mechanism for guanine alkylation by these chemical carcinogens. Moreover, it represents a good confirmation of the applicability of quantum chemical simulations to alkylation reactions related to carcinogenesis [12,16]. This encouraged us to employ the Hartree-Fock method with three different flexible basis sets to predict the activation barriers of chemical reactions involving the nine ultimate carcinogens and two natural scavengers, namely [6]-gingerol and glutathione, for which kinetic experiments have not been performed yet.

As biochemical reactions do not take place *in vacuo*, solvation effects had to be incorporated by the Self-consistent reaction field (SCRF) method of Tomasi and co-workers [38] and the Langevin dipoles (LD) model of Florian and Warshel [39]. The Merz-Kollman partial atomic charges obtained by applying the Gaussian 09 program suite at the corresponding HF level of theory served as the input for the LD model implemented in the ChemSol program [40]. In order to evaluate computational results, we compared the obtained activation free energies ∆G^⧧^ for the reactions of the studied chemical carcinogens with [6]-gingerol and glutathione to the experimental free energy barriers ∆G^⧧^ for the competing reactions of these chemical carcinogens with the most reactive DNA base guanine. The latter were obtained from the experimentally determined reaction rate constants k on the basis of the transition state theory of Eyring,

(1) $$k=\frac{k_{B}T}{h}e^{\left(-\frac{\Lambda G^{‡}}{k_{B}T}\right)}.$$

where k_B_ represents the Boltzmann constant, h the Planck constant, T the thermodynamic temperature, and ∆G^⧧^ the activation free energy. Transition state theory is based on the assumption that reactants and transition states form a thermal equilibrium [16].

Quantum-mechanical calculations were carried out on the CROW cluster located at the National Institute of Chemistry in Ljubljana [41]. The Born Oppenheimer hypersurfaces of the alkylation reactions of [6]-gingerol and glutathione with the nine studied chemical carcinogens were obtained by *ab initio* calculations at the Hartree-Fock level of theory with the Gaussian 09 suite of programs. HF method was used in conjunction with three flexible basis sets: 6-31G(d), 6-31+G(d,p), and 6-311++G(d,p).

In order to obtain the activation free energies of these reactions, we had to locate the corresponding reactant and transition state structures. For the starting reactant structures, we combined optimized structures for each of the reacting species ([6]-gingerol with the nine studied ultimate carcinogens and glutathione with the same nine ultimate carcinogens) so that the distances between the reacting atoms were around 3 Å. Than geometry optimizations were performed in order to obtain the structures lying in the local minimum of the potential energy surfaces. For the evaluation of the located structures, the vibrational analyses in the harmonic approximation were performed. The correctly optimized structures of the reactants must yield only real frequencies. The obtained reactant state structures were then subjected to a relaxed potential surface scan [16] to uncover approximate structures of the corresponding transition states. The obtained structures with the highest energy were chosen as the starting point for the Berny algorithm [42] that provided the optimized transition state structures. Subsequent vibrational analyses in the harmonic approximation were again performed. The correct transition state structures, lying in the first-order saddle points of the potential energy surface, have exactly one imaginary frequency that corresponds to the reaction coordinate, which represents the cleavage of one C-O epoxy bond within the ultimate chemical carcinogen and the formation of a new covalent bond connecting both reactants into the formed adduct. The activation free energy for this *S_N_2* reaction represents the free energy difference between the corresponding transition and reactant states.

## 4. Conclusions and Future Perspectives

We performed the first quantum mechanical simulations of reactions between two natural scavengers, namely [6]-gingerol and glutathione, and nine ultimate chemical carcinogens of the epoxy type. These alkylation reactions were considered from a kinetic standpoint by assessing the *ab initio* calculated activation free energies and comparing them to the experimentally determined ones.

The applied Hartree−Fock method in combination with the SCRF and LD implicit solvation models despite its limitations tends to offer results that compare favorably with the experimental activation free energies of alkylation reactions. With the selection of the right combination of the theory level (Hartree−Fock), flexible basis set (6-311++G(d,p)), and appropriate implicit solvation model (SCRF or LD), we should, therefore, obtain accurate predictions of absolute reactivities of the studied chemical carcinogens with [6]-gingerol and glutathione. The obtained results also present strong evidence in favor of the validity of the proposed *S_N_2* reaction mechanism and point to the applicability of quantum chemical methods to reactions related to chemical carcinogenesis. Moreover, our quantum mechanical study of the alkylation reactions of chemical carcinogens with [6]-gingerol and glutathione provide valuable insights into the reaction mechanisms and the geometries of the corresponding transition states. Finally, the results of our study potentially identified a novel natural scavenger, namely [6]-gingerol, that could effectively prevent DNA alkylation damage by covalently binding to the large majority of studied ultimate carcinogens of the epoxy type via a lower activation barrier than glutathione.

However, chemical reactivity is not the only parameter that may influence the scavenging potential of natural compounds in living cells. It is important to emphasize, that enzymatic detoxification reactions were not incorporated in our calculations. For example, glutathione transferases could decrease the activation free energies of conjugation reactions between glutathione and chemical carcinogens during the detoxification process [43]. Therefore, future *in vitro* studies, which could confirm the efficacy of [6]-gingerol to protect cells from the examined chemical carcinogens, are both envisaged and strongly encouraged. We firmly believe that our research represents the basis for further computational, experimental and clinical studies of anticarcinogenic properties of [6]-gingerol as well as for the development of novel chemoprophylactic dietary supplements.

## Figures and Tables

**Table 1:** The comparison of activation free energies for the reactions between nine studied ultimate chemical carcinogens and [6]-gingerol as well as glutathione at the HF/6-311++G(d,p) level of theory.

Method/Basis Set HF/6-311++G(d,p)	$\Delta E_{}^{‡}\;[\mathrm{kcal}/\mathrm{mol}]{\;}^{a}$	$\Delta \Delta G_{hydr}^{SCRF}[\mathrm{kcal}/\mathrm{mol}]{\;}^{b}$	$\Delta G_{SCRF}^{‡}\;[\mathrm{kcal}/\mathrm{mol}]{\;}^{c}$	*ω*^TS^ [i cm^−1^] *^d^*	*ω*^R^ [cm^−1^] *^e^*	*d*^TS^ [Å] *^f^*	*d*^R^ [Å] *^g^*
**Glycidamide**							
**[6]-Gingerol**	30.81	−5.58	25.23	646.37	6.99	1.91	3.18
**Glutathione**	35.50	−3.28	32.22	585.04	14.51	2.40	4.12
**Styrene Oxide**							
**[6]-Gingerol**	26.42	−3.76	21.65	647.91	2.34	1.96	3.19
**Glutathione**	30.23	−1.33	31.56	581.77	12.00	2.44	4.14
**AFB1 Exo-8.9-Epoxide**							
**[6]-Gingerol**	20.87	−5.49	15.38	317.15	7.02	2.21	3.62
**Glutathione**	17.56	−1.88	15.68	213.25	10.74	2.60	3.97
**Beta Propiolactone**							
**[6]-Gingerol**	16.50	−1.57	14.93	644.29	6.27	2.05	2.81
**Glutathione**	22.89	−0.38	22.51	614.21	14.72	2.55	3.85
**Ethylene Oxide**							
**[6]-Gingerol**	24.48	−0.45	24.03	629.98	8.29	1.95	3.26
**Glutathione**	30.63	−0.61	30.02	569.22	11.68	2.44	4.12
**Propylene Oxide**							
**[6]-Gingerol**	24.41	−0.04	24.37	615.67	7.58	1.94	3.28
**Glutathione**	31.01	−0.99	30.02	560.64	4.92	2.42	4.04
**2-Cyanoethylene Oxide**							
**[6]-Gingerol**	20.72	−0.45	20.27	677.50	7.67	1.98	3.07
**Glutathione**	26.36	−2.35	24.01	623.35	6.57	2.48	3.77
**Chloroethylene Oxide**							
**[6]-Gingerol**	21.10	−2.37	18.73	651.63	6.55	2.05	3.18
**Glutathione**	21.60	−0.02	21.59	627.92	14.19	2.57	3.91
**Vinyl Carbamate Epoxide**							
**[6]-Gingerol**	19.65	−0.18	18.97	620.03	5.70	2.06	3.17
**Glutathione**	25.28	−0.70	24.59	584.39	15.41	2.47	4.92

*^a^* Gas-phase activation energy. *^b^* Relative hydration free energy: hydration free energy of the transition state minus hydration free energy of the reactant state obtained by the Self-consistent reaction field (SCRF) method. *^c^* Activation free energy obtained by the SCRF method. *^d^* The imaginary frequency corresponding to the transition state. *^e^* The lowest frequency value corresponding to the reactant state. *^f^* The distance between the nucleophilic phenolic oxygen on [6]-gingerol or sulfur on glutathione and the nonchiral electrophilic carbon in the epoxy ring of the ultimate chemical carcinogen in the transition state structure. *^g^* The distance between the nucleophilic phenolic oxygen on [6]-gingerol or sulfur on glutathione and the nonchiral electrophilic carbon of the epoxy ring of the ultimate chemical carcinogen in the reactant state structure.

**Table 2:** Activation free energies for alkylation reactions of the studied chemical carcinogens with [6]-gingerol, glutathione and guanine obtained by the SCRF and LD implicit solvation models at the HF/6-311++G(d,p) level of theory.

Method/Basis Set HF/6-311++G(d,p)	[6]-Gingerol [kcal/mol]		Glutathione [kcal/mol]		Guanine [kcal/mol]		^a^ Experimental Value for Guanine [kcal/mol]
	$\Delta G_{SCRF}^{‡}{\;}^{a}$	$\Delta G_{LD}^{‡}{\;}^{b}$	$\Delta G_{SCRF}^{‡}{\;}^{a}$	$\Delta G_{LD}^{‡}{\;}^{b}$	$\Delta G_{SCRF}^{‡}{\;}^{a}$	$\Delta G_{LD}^{‡}$ ^b^	$\Delta G_{\exp}{\;}^{c}$
**Styrene Oxide**	21.65	26.03	31.56	30.16	-[4]	27.65 [4]	26.5 [4]
**Propylene Oxide**	24.37	23.57	30.02	26.06	21.26 [11]	25.15 [11]	25.4 [11]
**Ethylene Oxide**	24.03	21.99	30.02	24.17	21 [10]	24.62 [10]	24.7 [10]
**Glycidamide**	25.23	19.65	32.22	28.19	25.78 [1]	23.55 [1]	22.8 [1]
**Vinyl Carbamate Epoxide**	18.97	19.16	24.59	22.09	22.15 [16]	19.13 [16]	22.4 [16]
***β*-Propiolactone**	14.93	12.10	22.51	17.79	22.45	12.06	20.8 [37]
**Chloroethylene oxide**	18.73	17.57	21.59	18.78	17.26 [13]	22.87 [13]	19.5 [13]
**2-Cyanoethylene Oxide**	20.27	18.19	26.36	26.78	28.48 [12]	19.02 [12]	19.2 [12]
**AFB1 Exo-8.9-Epoxide**	15.38	5.44	15.68	5.21	18.9 [7]	14.25 [7]	15.1 [7]

^a^ Activation free energies of the alkylation reactions of the studied chemical carcinogens with [6]-gingerol, glutathione and guanine obtained by the SCRF implicit solvation model. ^b^ Activation free energies of the alkylation reactions of the studied chemical carcinogens with [6]-gingerol, glutathione and guanine obtained by the LD implicit solvation model. ^c^ Experimentally determined activation free energies for alkylation reactions between guanine and the studied chemical carcinogens. Based on the comparison of calculated and experimental activation free energies between studied chemical carcinogens and guanine, we predicted the calculated activation free energies that should be in a better agreement with experiments for [6]-gingerol and glutathione as well and underlined them.

## Supplementary Materials

Supplementary materials can be found at https://www.mdpi.com/1422-0067/21/3/695/s1.

## Abbreviations

AFB1	Aflatoxin B1
AP-1	Activator protein 1
BPL	Beta-propiolactone
CEO	Chloroethylene oxide
DNA	Deoxyribonucleic acid
ERK1/2	Extracellular signal–regulated kinase
ETO	Ethylene oxide
GSH	Glutathione
HF	Hartree−Fock level of theory
JNK	c-Jun N-terminal kinase
LD	Langevin dipoles implicit solvation model
MAPK	Mitogen-activated protein kinase
MMP-9	Matrix metalloproteinase 9
NADP	Nicotinamide adenine dinucleotide phosphate
NF- κB	Nuclear factor kappa B
PAHs	Polyaromatic hydrocarbons
PO	Propylene oxide
SCRF	Self-consistent reaction field implicit solvation model
STO	Styrene-7,8-oxide
TPA	Tetradecanoylphorbol-13-acetate
VC	Vinyl chloride

## Appendix A

The proposed *S_N_2* substitution mechanisms for the formation of the ultimate chemical carcinogen-[6]-gingerol adducts are depicted in Scheme A1, Scheme A2, Scheme A3, Scheme A4, Scheme A5, Scheme A6, Scheme A7, Scheme A8 and Scheme A9. Similarly, the *S_N_2* substitution mechanisms are proposed for the formation of ultimate chemical carcinogen-glutathione adducts, which are presented in Scheme A1, Scheme A2, Scheme A3, Scheme A4, Scheme A5, Scheme A6, Scheme A7, Scheme A8 and Scheme A9 as well.

The phenolic oxygen of [6]-gingerol or the nucleophilic sulfur of glutathione attacks the electrophilic nonchiral epoxy carbon of the ultimate chemical carcinogen. An unstable anionic intermediate is formed, which is followed by subsequent protonation of the epoxy oxygen in the cases of glycidamide, beta-propiolactone, AFB1 exo-8,9-epoxide, ethylene oxide, propylene oxide, and styrene oxide or by the elimination of the good leaving group in the cases of 2-cyanoethylene oxide, chloroethylene oxide and vinyl carbamate epoxide. The *S_N_2* substitution represents the rate-limiting step of these reactions.
