# Supplementary material for: Protective Effects of [6]-Gingerol Against Chemical Carcinogens: Mechanistic Insights
Source: Int J Mol Sci. 2020 Jan 21;21(3):695. doi: 10.3390/ijms21030695 (PMC7037038; doi:10.3390/ijms21030695)
Supplement: Supplementary file 1 [file ijms-21-00695-s001.pdf]

Supplementary Materials

Protective Effects of [6]-Gingerol against Chemical Carcinogens: Mechanistic Insights

Veronika Furlan[1], Urban Bren[1,2,*]

[1] Faculty of Chemistry and Chemical Technology, University of Maribor, Smetanova 17, SI-2000 Maribor, Slovenia
[2] National Institute of Chemistry, Hajdrihova 19, SI-1001 Ljubljana, Slovenia
* Correspondence: urban.bren@um.si; Tel.: +386-2-229 4421

The calculated activation barriers at the Hartree-Fock level of theory in conjunction with three flexible basis sets for the alkylation reactions of the nine studied ultimate chemical carcinogens with [6]-gingerol and glutathione *in vacuo* and solvated with SCRF or LD methods, imaginary frequencies of transition states, lowest vibrational frequencies of reactant states and the corresponding distances between the reactive centers are collected in Table S1.

Table S1 The comparison of activation free energies for the reactions of nine studied ultimate chemical carcinogens with [6]-gingerol and glutathione.

Method/Basis set		$\Delta E^{\ddagger}$ [kcal/mol][a]	$\Delta\Delta G_{hydr}^{SCRF}$ [kcal/mol][b]	$\Delta G_{SCRF}^{\ddagger}$ [kcal/mol][c]	$\Delta\Delta G_{hydr}^{LD}$ [kcal/mol][d]	$\Delta G_{LD}^{\ddagger}$ [kcal/mol][e]	ω^{TS} [i cm^{-1}][f]	ω^{R} [cm^{-1}][g]	d^{TS} [Å][h]	d^{R} [Å][i]
**Glycidamide**										
[6]-gingerol	HF/6-31G(d)	31,32	-12,41	18,91	-8,48	22,84	657,70	5,54	1,88	3,15
	HF/6-31+G(d,p)	30,22	-5,60	24,62	-11,22	19,00	643,00	6,86	1,91	3,18
	HF/6-311++G(d,p)	30,81	-5,58	25,23	-11,16	19,65	646,37	6,99	1,91	3,18
Glutathione	HF/6-31G(d)	38,83	-2,31	36,52	-5,98	32,85	567,03	14,33	2,34	5,12
	HF/6-31+G(d,p)	35,71	-3,51	32,20	-7,69	28,02	574,83	14,59	2,38	5,12
	HF/6-311++G(d,p)	35,50	-3,28	32,22	-7,31	28,19	585,04	14,51	2,40	5,12
**Styrene oxide**										
[6]-gingerol	HF/6-31G(d)	26,52	-3,73	21,38	-1,41	25,11	664,84	3,70	1,93	3,19
	HF/6-31+G(d,p)	26,43	-4,12	21,09	-1,22	25,21	647,15	3,48	1,96	3,20
	HF/6-311++G(d,p)	26,42	-3,76	21,65	-0,39	26,03	647,91	2,34	1,96	3,19
Glutat	HF/6-31G(d)	33,27	-2,45	35,73	-0,11	33,16	568,00	12,93	2,39	4,07
	HF/6-31+G(d,p)	30,32	-1,24	31,56	-1,03	29,29	570,31	12,13	2,42	4,12

	HF/6-311++G(d,p)	30,23	-1,33	31,56	-0,07	30,16	581,77	12,00	2,44	4,14

	Method/Basis set	$\Delta E^{\ddagger}$ [kcal/mol][a]	$\Delta\Delta G_{hydr}^{SCRF}$ [kcal/mol][b]	$\Delta G_{SCRF}^{\ddagger}$ [kcal/mol][c]	$\Delta\Delta G_{hydr}^{LD}$ [kcal/mol][d]	$\Delta G_{LD}^{\ddagger}$ [kcal/mol][e]	ω^{TS} [i cm^{-1}][f]	ω^{R} [cm^{-1}][g]	d^{TS} [Å][h]	d^{R} [Å][i]
**AFB1 exo-8,9-epoxide**										
**[6]-gingerol**	HF/6-31G(d)	18,14	-2,25	15,89	-13,18	4,96	429,20	2,58	2,11	3,04
	HF/6-31+G(d,p)	20,96	-5,56	15,39	-15,38	5,58	331,34	7,02	2,20	3,62
	HF/6-311++G(d,p)	20,87	-5,49	15,38	-15,43	5,44	317,15	7,02	2,21	3,62
**Glutathione**	HF/6-31G(d)	21,44	-5,60	15,84	-16,26	5,18	284,73	8,04	2,50	4,02
	HF/6-31+G(d,p)	17,75	-1,92	15,82	-12,56	5,19	223,27	11,74	2,58	3,96
	HF/6-311++G(d,p)	17,56	-1,88	15,68	-12,35	5,21	213,25	10,74	2,60	3,97
**Beta propiolactone**										
**[6]-gingerol**	HF/6-31G(d)	15,82	-1,62	14,19	-3,82	12,00	657,50	6,27	2,02	2,78
	HF/6-31+G(d,p)	15,47	-1,62	13,85	-4,14	11,33	639,68	5,93	2,05	2,80
	HF/6-311++G(d,p)	16,50	-1,57	14,93	-4,4	12,10	644,29	6,27	2,05	2,81
**Glutathione**	HF/6-31G(d)	24,22	-0,06	24,16	-3,63	20,59	588,89	14,16	2,52	3,73
	HF/6-31+G(d,p)	22,45	-0,48	21,97	-5,07	17,38	602,97	14,97	2,54	3,82
	HF/6-311++G(d,p)	22,89	-0,38	22,51	-5,1	17,79	614,21	14,72	2,55	3,85
**Ethylene oxide**										
**[6]-gingerol**	HF/6-31G(d)	24,76	-0,14	24,61	-1,98	22,78	645,82	2,88	1,91	2,99
	HF/6-31+G(d,p)	23,80	-0,36	23,44	-1,65	22,15	629,84	8,21	1,95	3,26
	HF/6-311++G(d,p)	24,48	-0,45	24,03	-2,49	21,99	629,98	8,29	1,95	3,26
**Glutathione**	HF/6-31G(d)	35,13	-1,66	33,48	-6,17	28,96	558,37	12,41	2,38	3,93
	HF/6-31+G(d,p)	30,37	-0,27	30,10	-6,14	24,23	560,29	11,92	2,42	4,09
	HF/6-311++G(d,p)	30,63	-0,61	30,02	-6,46	24,17	569,22	11,68	2,44	4,12
**Propylene oxide**										
**[6]-gingerol**	HF/6-31G(d)	25,47	-0,70	24,77	-1,58	23,89	632,42	8,10	1,90	3,31
	HF/6-31+G(d,p)	24,03	-0,29	23,74	-1,11	22,92	615,87	7,48	1,94	3,27
	HF/6-311++G(d,p)	24,41	-0,04	24,37	-0,84	23,57	615,67	7,58	1,94	3,28
**Glutathione**	HF/6-31G(d)	35,83	-4,81	31,01	-3,82	32,01	547,03	6,15	2,37	3,80
	HF/6-31+G(d,p)	31,12	-1,02	30,09	-5,44	25,68	550,06	6,32	2,41	4,01
	HF/6-311++G(d,p)	31,01	-0,99	30,02	-4,95	26,06	560,64	4,92	2,42	4,04

	Method/Basis set	$\Delta E^{\ddagger}$ [kcal/mol][a]	$\Delta\Delta G_{hydr}^{SCRF}$ [kcal/mol][b]	$\Delta G_{SCRF}^{\ddagger}$ [kcal/mol][c]	$\Delta\Delta G_{hydr}^{LD}$ [kcal/mol][d]	$\Delta G_{LD}^{\ddagger}$ [kcal/mol][e]	ω^{TS} [i cm^{-1}][f]	ω^{R} [cm^{-1}][g]	d^{TS} [Å][h]	d^{R} [Å][i]
**2-Cyanoethylene oxide**										
**[6]-gingerol**	HF/6-31G(d)	20,20	-0,56	19,64	-1,41	18,76	686,53	7,51	1,95	3,18
	HF/6-31+G(d,p)	19,85	-0,35	19,50	-1,74	18,11	673,67	7,98	1,98	3,08
	HF/6-311++G(d,p)	20,72	-0,45	20,27	-2,53	18,19	677,50	7,67	1,98	3,07
**Glutathione**	HF/6-31G(d)	26,46	-2,17	24,29	-0,59	25,87	597,24	6,74	2,44	3,76
	HF/6-31+G(d,p)	26,27	-2,41	23,85	0,41	26,68	612,30	7,03	2,47	3,76
	HF/6-311++G(d,p)	26,36	-2,35	24,01	0,42	26,78	623,35	6,57	2,48	3,77
**Chloroethylene oxide**										
**[6]-gingerol**	HF/6-31G(d)	21,46	-2,55	18,91	-3,77	17,69	678,93	7,54	2,03	3,18
	HF/6-31+G(d,p)	20,87	-2,51	18,37	-4,34	16,53	653,27	6,73	2,05	3,18
	HF/6-311++G(d,p)	21,10	-2,37	18,73	-3,53	17,57	651,63	6,55	2,05	3,18
**Glutathione**	HF/6-31G(d)	23,81	-0,65	23,15	-3,5	20,31	635,87	12,52	2,54	3,85
	HF/6-31+G(d,p)	21,70	-0,09	21,61	-3,92	17,78	619,60	14,04	2,56	3,89
	HF/6-311++G(d,p)	21,60	-0,02	21,59	-2,82	18,78	627,92	14,19	2,57	3,91
**Vinyl carbamate epoxide**										
**[6]-gingerol**	HF/6-31G(d)	18,65	-0,06	18,59	-0,26	18,39	634,50	6,36	2,05	3,17
	HF/6-31+G(d,p)	18,44	-0,08	18,36	-0,25	18,19	617,63	5,57	2,06	3,16
	HF/6-311++G(d,p)	19,65	-0,18	18,97	-0,49	19,16	620,03	5,70	2,06	3,17
**Glutathione**	HF/6-31G(d)	25,36	-6,01	19,36	-3,28	22,08	562,09	3,14	2,41	3,77
	HF/6-31+G(d,p)	24,96	-0,71	24,26	-4,16	20,80	572,84	15,60	2,46	4,91
	HF/6-311++G(d,p)	25,28	-0,70	24,59	-3,19	22,09	584,39	15,41	2,47	4,92

[a]Gas-phase activation energy. [b] Relative hydration free energy: hydration free energy of the transition state minus hydration free energy of the reactant state obtained by the SCRF method. [c] Activation free energy obtained by the SCRF method. [d] Relative hydration free energy: hydration free energy of the transition state minus hydration free energy of the reactant state obtained by the LD method. [e] Activation free energy obtained by the LD method. [f] The imaginary frequency corresponding to the transition state. [g] The lowest frequency value corresponding to the reactant state. [h] The distance between the phenolic oxygen on [6]-gingerol or sulfur on glutathione and the electrophilic nonchiral carbon in the epoxy ring of the ultimate chemical carcinogen in the transition state structure. [i] The distance between the phenolic oxygen on [6]-gingerol or sulfur on glutathione and the electrophilic nonchiral carbon in the epoxy ring of the ultimate chemical carcinogen in the reactant state structure.

In Table S2 the comparison of activation free energies for the reactions of [6]-gingerol and glutathione with the nine studied chemical carcinogens obtained at the HF/6-311++G(d,p) and M06-2X/6-311++G(d,p) levels of theory in conjunction the SCRF and LD implicit solvation models is presented.

Table S2 The comparison of activation free energies for the reactions of the nine studied ultimate chemical carcinogens with [6]-gingerol and glutathione obtained at the HF/6-311++G(d,p) and M06-2X/6-311++G(d,p) level of theory in conjunction with the SCRF and LD implicit solvation models.

Method/Basis set	[6]-Gingerol [kcal/mol]		Glutathione [kcal/mol]		[6]-Gingerol [kcal/mol]		Glutathione [kcal/mol]	
	HF/6-311++G(d,p)				M06-2X/6-311++G(d,p)			
	$\Delta G^{\ddagger}_{SCRF}$ a	$\Delta G^{\ddagger}_{LD}$ b	$\Delta G^{\ddagger}_{SCRF}$ a	$\Delta G^{\ddagger}_{LD}$ b	$\Delta G^{\ddagger}_{SCRF}$ a	$\Delta G^{\ddagger}_{LD}$ b	$\Delta G^{\ddagger}_{SCRF}$ a	$\Delta G^{\ddagger}_{LD}$ b
**Styrene oxide**	21.65	26.03	31.56	30.16	20.32	23.71	26.27	25.81
**Propylene oxide**	24.37	23.57	30.02	26.06	22.35	20.47	28.64	25.19
**Ethylene oxide**	24.03	21.99	30.02	24.17	23.91	21.02	26.94	22.57
**Glycidamide**	25.23	19.65	32.22	28.19	24.91	18.46	29.25	27.81
**Vinyl carbamate epoxide**	18.97	19.16	24.59	22.09	16.68	15.98	19.39	18.69
***β*-propiolactone**	14.93	12.10	22.51	17.79	14.16	13.13	19.66	17.41
**Chloroethylene oxide**	18.73	17.57	21.59	18.78	15.64	15.45	18.52	17.98
**2-cyanoethylene oxide**	20.27	18.19	26.36	26.78	20.17	16.73	21.47	20.37
**AFB1 exo-8.9-epoxide**	15.38	5.44	15.68	5.21	14.26	5.37	15.05	5.13

The calculated activation free energies with Minessota functional M06-2X, which is currently very popular in computational chemistry, are significantly lower than the activation free energies obtained at the Hartree-Fock level of theory. However, the trend of reactivity remains the same. In our previous studies was shown, that the activation free energies obtained at the Hartree-Fock level of theory in conjunction with 6-311++G(d,p) flexible basis set yield very good agreement with experimental activation free energies for alkylation reactions.[1-5] Therefore, the Hartree-Fock level of theory in conjunction with 6-311++G(d,p) flexible basis set and implicit solvation model was primarily used to predict the activation free energies for alkylation reactions of chemical carcinogens with [6]-gingerol and glutathione.

References

1. Bren, U.; Guengerich, F. P.; Mavri, J., Guanine alkylation by the potent carcinogen aflatoxin B1: quantum chemical calculations. *Chem. Res. Toxicol.* **2007,** *20(8)*, 1134-1140.
2. Bren, U.; Zupan, M.; Guengerich, F. P.; Mavri, J., Chemical reactivity as a tool to study carcinogenicity: reaction between chloroethylene oxide and guanine. *J. Org. Chem.* **2006,** *71(11)*, 4078-4084.
3. Galesa, K.; Bren, U.; Kranjc, A.; Mavri, J., Carcinogenicity of acrylamide: a computational study. *J. Agric. Food. Chem.* **2008,** *56(18)*, 8720-8727.
4. Lajovic, A.; Nagy, L. D.; Guengerich, F. P.; Bren, U., Carcinogenesis of urethane: simulation versus experiment. *Chem. Res. Toxicol.* **2015,** *28(4)*, 691.
5. Gladović, M.; Španinger, E.; Bren, U., Nucleic bases alkylation with acrylonitrile and cyanoethylene oxide: A computational study. *Chem. Res. Toxicol.* **2017,** *31(2),* 97-104.
